# Tentonin 3 regulates the proliferation and migration of neural stem cells during embryonic brain development

**DOI:** 10.1186/s12915-026-02604-9

**Published:** 2026-04-21

**Authors:** Maria Al Nuilati, Kyungmin Kim, Jimin Kang, Min Soo Kim, Uhtaek Oh, Gyu-Sang Hong

**Affiliations:** 1 Brain Science Institute, Korea Institute of Science and Technology (KIST), Seoul, 02792 Republic of Korea; 2 Division of Bio-Medical Science & Technology, University of Science and Technology KIST School, Seoul, 02792 Republic of Korea; 3 Department of Molecular Medicine and Biopharmaceutical Sciences, Graduate School of Convergence Science and Technology, Seoul National University, Seoul, 08826 Republic of Korea

**Keywords:** Tentonin 3, Mechanosensitive ion channel, Brain development, Embryogenesis, Mechanobiology, Neural stem cells

## Abstract

**Background:**

Cell fate determination during brain development, encompassing both neural stem cells (NSCs) and immature neurons, is orchestrated by complex mechanical signals. While mechanical sensing during forebrain development is gaining recognition, the underlying regulatory genes remain underexplored.

**Results:**

In this study, we present a comprehensive analysis of the expression patterns of the mechanosensitive channel Tentonin 3 (TMEM150C, TTN3) across critical stages of embryonic brain development (E12.5, E14.5, E16.5, and E18.5) using RNAscope technology. We investigated *Ttn3* expression in NSCs and immature neurons through co-staining with *Nestin, Eomesodermin,* and *Doublecortin* probes. *Ttn3* transcripts were detected across multiple embryonic brain regions and layers, with a higher expression in the superficial layers compared to the ventricular and subventricular zones. In addition, we demonstrate that TTN3 is involved in the proliferation and migration of progenitor cells.

**Conclusions:**

Our findings suggest that TTN3 may play a critical role in embryonic brain development, potentially contributing as a mechanotransduction mediator.

**Supplementary Information:**

The online version contains supplementary material available at 10.1186/s12915-026-02604-9.

## Background

During embryogenesis, neural stem cell (NSC) undergoes an expansion phase, initially characterized by symmetric divisions that yield additional progenitor progeny [1]. As neurogenesis commences, NSC switches from symmetric to asymmetric divisions, producing one NSC and one differentiating daughter cell [1]. The processes of neurogenesis and gliogenesis involve systematic proliferation and differentiation of neural progenitors alongside tightly coordinated radial and tangential migration [2, 3]. These events are orchestrated not only by chemical signals but also by mechanical cues, including tissue stiffness, hydrostatic pressure, shear flow, and cytoskeletal tension [4]. Such mechanosensitive (MS) inputs critically influence cell fate decisions [5, 6]. The mechanosensory ability of these cells is primarily mediated by mechanotransduction, a process where mechanical signals from the extracellular matrix (ECM) are converted into biochemical responses [7]. In particular, MS channels play a pivotal role in this process by directly facilitating Ca^2+^ influx in response to mechanical stimuli.

In the developing mouse brain, the expression patterns of Piezo1, a rapidly-inactivating MS channel, have been characterized [8, 9]. Notably, its inhibition has been shown to enhance cell migration, highlighting its regulatory role in this process [8, 10]. *Piezo1* knockout (KO) mice exhibit a thinner neuroepithelial layer, while *Piezo1* silencing suppresses neurogenesis and promotes astrogenesis, further demonstrating its critical role in brain development [11, 12]. Despite this, the relatively limited expression of *Piezo1* in the fetal brain suggests the involvement of additional MS channels during embryogenesis [8]. Tentonin 3/TMEM150C (TTN3) has been recently identified as a pore-forming MS channel [13–15]. TTN3 mediates slowly adapting MS currents in sensory neurons, contributing to muscle coordination and arterial pressure regulation [13, 16]. Loss of *Ttn3* has been associated with impaired mechanotransduction and glucose tolerance in pancreatic β-cells [17]. Given these functions, TTN3's expression patterns may hold key insights into its potential role in brain development.

This study aimed to investigate the expression patterns of TTN3 in the developing mouse brain at key embryonic stages—E12.5, E14.5, E16.5, and E18.5—utilizing fluorescence in situ hybridization (FISH). Additionally, we explored the role of TTN3 in regulating cell migration and proliferation in neurosphere-derived cells and embryos derived from TTN3 KO mice, providing insights into its potential contributions to embryonic brain development.

## Results

### *Ttn3* expression patterns in E12.5 developing mouse brain

To examine the expression of *Ttn3* across different cellular populations, we performed in situ staining for *Ttn3* (red) in combination with either NSC marker *Nestin* (green) or neuronal marker *Doublecortin* (*Dcx*) (green).

**Fig. 1:**
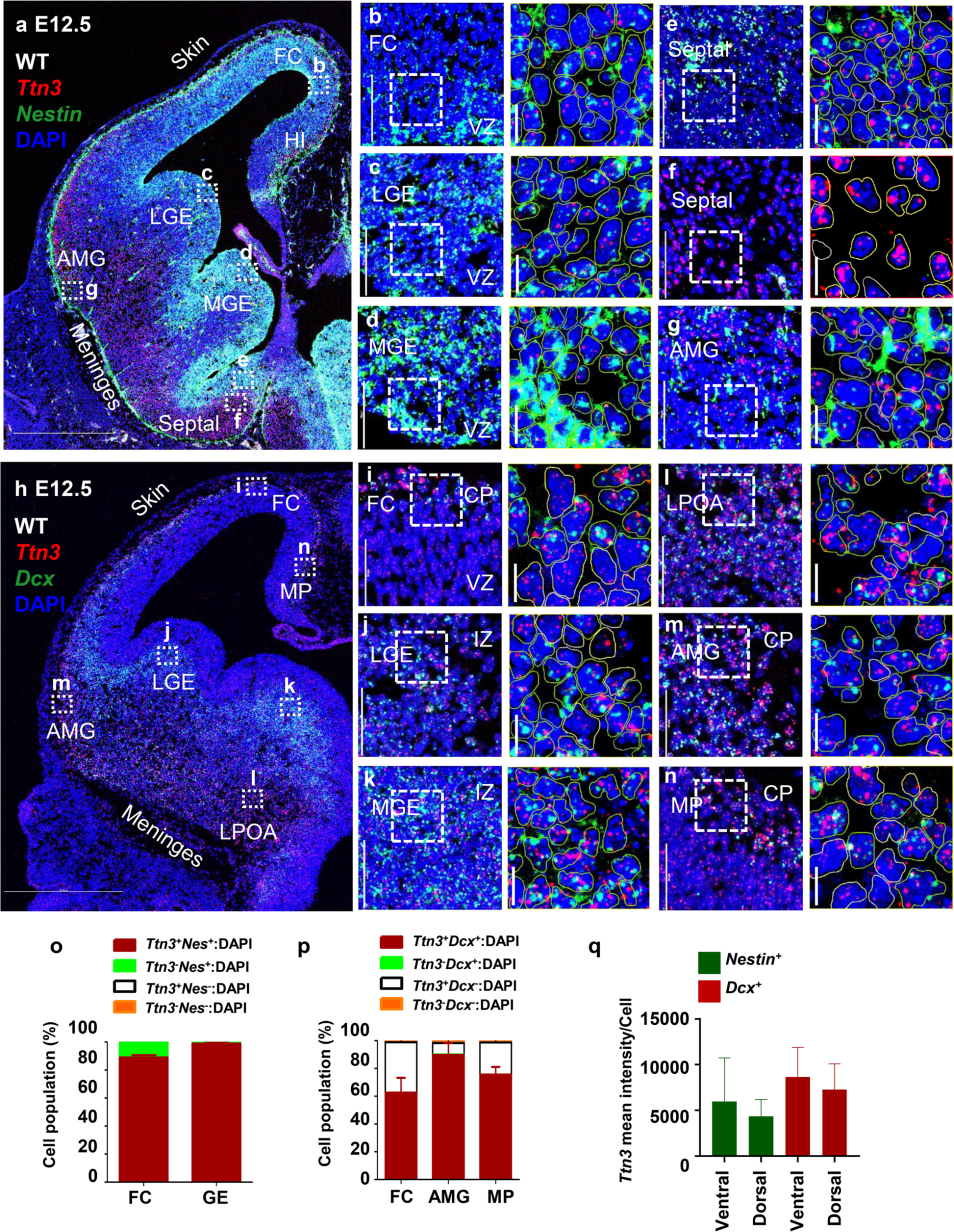
Expression patterns of *Ttn3* with *Nestin* and *Dcx* during forebrain development at E12.5. **a, h** Tile scans of an E12.5 mouse forebrain section illustrating *Ttn3* (red) expression with *Nestin* (green) (**a**) or *Dcx* (green) (**h**), co-stained with DAPI (blue) to label nuclei. Labeled regions include the FC, LGE, MGE, septal, and AMG providing an overview of *Ttn3*, *Nestin*, and *Dcx* distribution. **b–g, i–n** High-magnification (40X) images from specific brain. Specific areas imaged include: VZ in the FC (**b**), VZ in the LGE (**c**), VZ in the MGE (**d**), VZ in septal (**e**), IZ in the septal (**f**), CP in the AMG (**g**). CP and VZ in the FC (**i**), IZ in the LGE region (**j**), IZ in the MGE (**k**), LPOA region (**l**), CP in the AMG (**m**), CP in the MP (**n**). **o, p** Quantitative analysis of cell populations expressing *Ttn3* and *Nestin* or *Ttn3* and *Dcx*: **o** Summary of cell counts in the VZ of the FC and GE at E12.5, based on *Nestin* (green) and *Ttn3* (white) expression. Cells were classified into four groups: *Nestin*⁺:*Ttn3*⁺ (red), *Nestin*⁺:*Ttn3*⁻ (green), *Nestin*⁻:*Ttn3*⁺ (white), and *Nestin*⁻:*Ttn3*⁻ (orange). **p** Summary of cell counts in the CP regions of the FC, AMG, and MP at E12.5, based on *Dcx* (green) and *Ttn3* (white) expression. Cells were categorized into four groups: *Dcx*⁺:*Ttn3*⁺ (red), *Dcx*⁺:*Ttn3*⁻ (green), *Dcx*⁻:*Ttn3*⁺ (white), and *Dcx*⁻:*Ttn3*⁻ (orange). **q** *Ttn3* intensity analysis in *Nestin⁺* (Green) and *Dcx⁺* (Red) cells within the dorsal and ventral pallium. Abbreviation: FC, frontal cortex; LGE, lateral ganglionic eminence; MGE, medial ganglionic eminence; AMG, amygdala; LPOA, lateral preoptic area; MP, medial pallium. Scale bars: **a, h** 500 µm; **b–g, i–n** 50 µm; Cropped image 10 µm. All quantitative analyses were performed using *n* = 3 mice per group**.** Individual data values are provided in Additional file 4

At E12.5, *Nestin* transcripts were primarily localized in the ventricular zone (VZ), subventricular zone (SVZ), and a thin layer corresponding to the meninges at the cortical surface (Fig. 1a), indicating the presence of Nestin-expressing cells in the meningeal compartment [18], although their precise identity remains to be determined. *Ttn3* exhibited widespread expression at E12.5, with higher levels observed in the superficial layers of the cortex, amygdala (AMG), and Septal (Fig. 1a). Closer examination of wild-type (WT) magnified images revealed distinct localization of *Ttn3* transcripts within *Nestin*-positive (*Nestin*⁺) DAPI boundaries across several brain regions. These include the VZ of the frontal cortex (FC, Fig. 1b), as well as the VZ layer of Lateral ganglionic eminence (LGE) (Fig. 1c), Medial ganglionic eminence (MGE) (Fig. 1d), and the septal region (Fig. 1e, f). In the septal region, *Ttn3* was expressed in both *Nestin⁺* and *Nestin⁻* cells, with notably higher expression in *Nestin⁻* cells (Fig. 1e, f). In the amygdala region's neuronal layer, robust *Ttn3* expression was observed in *Nestin*^–^ cells (Fig. 1g).

Additionally, we performed co-staining with *Eomesodermin* (*Eomes*), a marker for cortical intermediate progenitor (IPs) (Additional file 1: Fig. S1a–c). *Ttn3* exhibited broad expression throughout the entire *Eomes*^+^ SVZ region as well as the neuronal layer rather than being restricted to specific IPs populations (Additional file 1: Fig. S1b, c). This *Ttn3* expression pattern was markedly reduced in TTN3 KO embryos (Additional file 2: Fig. S2a-g). A low level of residual RNAscope signal was occasionally observed, which likely reflects probe binding to transcript regions shared across multiple *Ttn3* isoforms that are not disrupted in the KO allele. *Dcx* transcripts were detected in the thin neuronal layer (a superficial layer) at E12.5, where immature neurons predominantly reside at this stage of embryonic development (Fig. 1h). Magnified images of these neuronal layers showed that *Ttn3* signals were notably higher in *Dcx*⁺ cells of FC (Fig. 1i), LGE (Fig. 1j), MGE (Fig. 1k), LPOA (Fig. 1l), AMG (Fig. 1m), and medial pallium (MP) (Fig. 1n). Cell population analysis further confirmed widespread *Ttn3* expression across multiple regions, including the cortex, AMG, and MP. For cell counting, unless otherwise noted, a total of nine sections (three per mouse from three distinct mice) were analyzed. Over 80% of cells in the cortical VZ and more than 98% of cells in the VZ of the GE were positive for *Ttn3* (Fig. 1o). Among these, approximately 88.7% of *Nestin⁺* cells in the cortex and nearly 99% in the GE expressed *Ttn3* (Fig. 1o). Notably, a small subset of *Nestin*⁺ cells, comprising approximately 11% in the cortical VZ, did not express *Ttn3* (Fig. 1o). In the cortical plate (CP), *Ttn3* was detected in over 99% of cortical cells, more than 97% of the AMG cells, and above 98% of cells of MP. *Ttn3* was also highly expressed in nearly all *Dcx*⁺ cells across these regions, with near-total co-expression: cortex (100%), AMG (approximately 99.6%), and MP (98%) (Fig. 1p).

Furthermore, to compare regional and laminar differences in *Ttn3* expression, we performed intensity analysis in both *Nestin⁺* and *Dcx⁺* cells across the dorsal and ventral pallium. Across both regions, the mean *Ttn3* intensity did not differ significantly between *Dcx*⁺ and *Nestin*⁺ cells (Fig. 1q).

### *Ttn3* expression patterns in E14.5 developing mouse brain

**Fig. 2:**
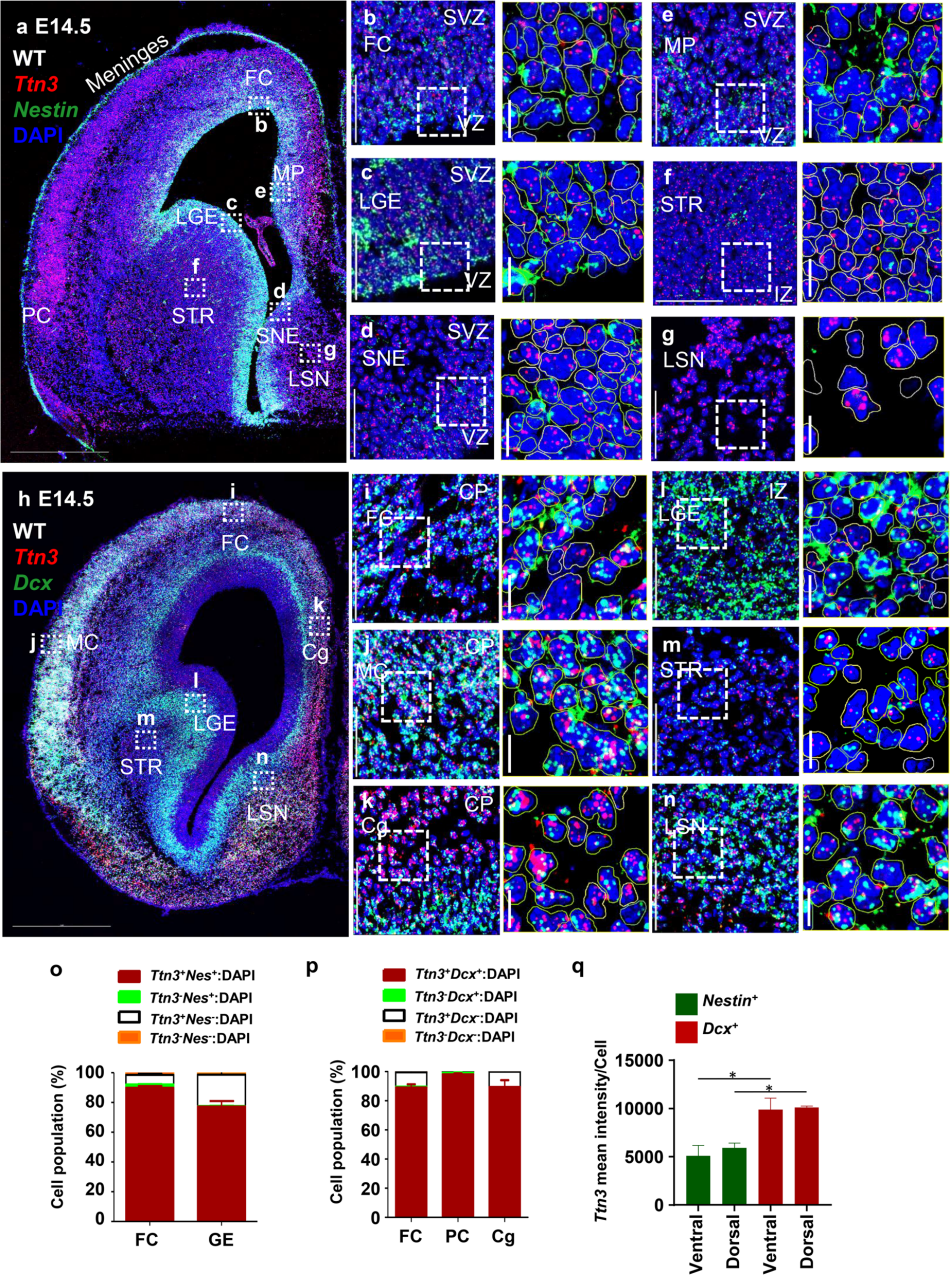
Expression patterns of *Ttn3* with *Nestin* and *Dcx* during forebrain development at E14.5. **a, h** Tile scans of an E14.5 mouse forebrain section illustrating *Ttn3* (red) expression with *Nestin* (green) (**a**) or *Dcx* (green) (**h**), co-stained with DAPI (blue) to label nuclei. Labeled regions include the FC, LGE, SNE, MP, STR, and LSN providing an overview of *Ttn3*, *Nestin*, and *Dcx* distribution. **b–g, i–n** High-magnification (40 ×) images from specific brain regions. Specific areas imaged include: VZ and SVZ in the FC (**b**), VZ and SVZ in the LGE (**c**), VZ and SVZ in the SNE (**d**), VZ and SVZ in the MP (**e**), IZ in STR (**f**), IZ in the LSN (**g**), CP in the FC (**i**), CP in the MC (**j**), CP in the Cg (**k**), IZ in LGE (**l**), STR (**m**), LSN (**n**). **o, p** Quantitative analysis of cell populations expressing *Ttn3* and *Nestin* or *Ttn3* and *Dcx*: **o** Summary of cell counts in the VZ region of the GE and cortex at E14.5, based on *Nestin* (green) and *Ttn3* (white) expression. Cells were classified into four groups: *Nestin*⁺: *Ttn3*⁺ (red), *Nestin*⁺: *Ttn3*⁻ (green), *Nestin*⁻: *Ttn3*⁺ (white), and *Nestin*⁻: *Ttn3*⁻ (orange). **p** Summary of cell counts in the superficial CP regions of the FC, PC, and Cg at E14.5, based on *Dcx* (green) and *Ttn3* (white) expression. Cells were categorized into four groups: *Dcx*⁺: *Ttn3*⁺ (red), *Dcx*⁺: *Ttn3*⁻ (green), *Dcx*⁻: *Ttn3*⁺ (white), and *Dcx*⁻: *Ttn3*⁻ (orange). **q** *Ttn3* intensity analysis in *Nestin⁺* (Green) and *Dcx⁺* (Red) cells within the dorsal and ventral pallium (*P < 0.05, **P < 0.01, ***P < 0.001; statistical significance determined by one-way ANOVA followed by Tukey’s post hoc test). Abbreviation: FC frontal cortex; LGE lateral ganglionic eminence; MGE medial ganglionic eminence; STR striatum; SNE septal neuroepithelium; LSN lateral septal nucleus, MP medial pallium, PC piriform cortex, Cg cingulate cortex. Scale bars: **a, h** 500 µm; **b–g, i–n** 50 µm; Cropped image 10 µm. All quantitative analyses were performed using *n* = 3 mice per group**.** Individual data values are provided in Additional file 4

At E14.5, we analyzed the expression patterns of *Ttn3* alongside *Nestin*. As neurogenesis progresses, *Nestin* expression becomes increasingly restricted to the VZ and SVZ, particularly in RG, which play a pivotal role in supporting neuronal migration and cortical organization. Consistent with expectations, *Nestin* expression was more localized to the VZ at E14.5 (Fig. 2a). Similar to its distribution at E12.5, *Ttn3* exhibited widespread expression across multiple brain regions, including the FC, medial cortex (MC), cingulate cortex (Cg), septal nucleus (SN), striatum (STR), MP, and LGE (Fig. 2a, h). However, at E14.5, *Ttn3* expression showed increased intensity compared to E12.5, with a more prominent localization in the superficial layers relative to the VZ and SVZ (Fig. 2a). High-resolution imaging revealed *Ttn3* expression in both *Nestin*⁺ and *Nestin*⁻ cells within the VZ and SVZ of regions such as the neuroepithelium layer of FC (Fig. 2b), LGE (Fig. 2c), septal neuroepithelium (SNE) (Fig. 2d), and MP (Fig. 2e), as well as in the intermediate zone (IZ) of the STR (Fig. 2f). While the VZ and SVZ exhibited comparable *Ttn3* intensity, immature neuronal layers in the lateral septal nucleus (LSN) showed significantly higher expression (Fig. 2g). Furthermore, co-staining with *Eomes* confirmed widespread *Ttn3* expression not only in *Eomes*⁺ IPs but throughout the VZ and SVZ (Additional file 1: Fig. S1d-f).

To further explore *Ttn3* expression patterns in the CP, we examined its co-expression with *Dcx*. At E14.5, there was an increase in *Dcx*⁺ migrating cells, reflecting the active movement of neurons to their final destinations during brain development. These cells were predominantly found in the IZ, subplate (SP), and CP—regions enriched with glutamatergic neurons, interneurons and IPs [19]. Consistent with expectations, *Ttn3* expression increased alongside the rise in *Dcx*⁺ cells (Fig. 2h). Magnified images revealed substantial co-expression of *Ttn3* in *Dcx*⁺ cells (Fig. 2i-n). Notably, *Dcx*⁺ cells in the CP layers of FC (Fig. 2i), MC (Fig. 2j), and Cg (Fig. 2k) exhibited higher *Ttn3* expression than those in the IZ layer of LGE (Fig. 2l), STR (Fig. 2m), and LSN (Fig. 2n) indicating a region-specific pattern of *Ttn3* distribution.

Cell population analysis confirmed that over 96% of cells in the cortical VZ and more than 98% of cells in the VZ of GE were *Ttn3*^+^ (Fig. 2o). In the GE region, nearly all *Nestin*^+^ cells (99.6%) expressed *Ttn3*, while 97.3% of *Nestin*^+^ cells in the cortical VZ were *Ttn3*^+^ (Fig. 2o). In the superficial layers, *Ttn3* was detected in over 99% of frontal cortical cells, piriform cortex (PC) cells, and Cg cells. Additionally, nearly all *Dcx*^+^ cells expressed *Ttn3,* with the following distribution: cortex (99.5%), PC (97.3%), Cg (100%) (Fig. 2p).

We further quantified regional *Ttn3* expression by measuring signal intensity in *Nestin*⁺ and *Dcx*⁺ cells within the dorsal and ventral pallium. In both regions, *Dcx⁺* cells exhibited higher *Ttn3* expression than *Nestin⁺* cells (Fig. 2q).

### *Ttn3* expression patterns in E16.5 developing mouse brain

**Fig. 3:**
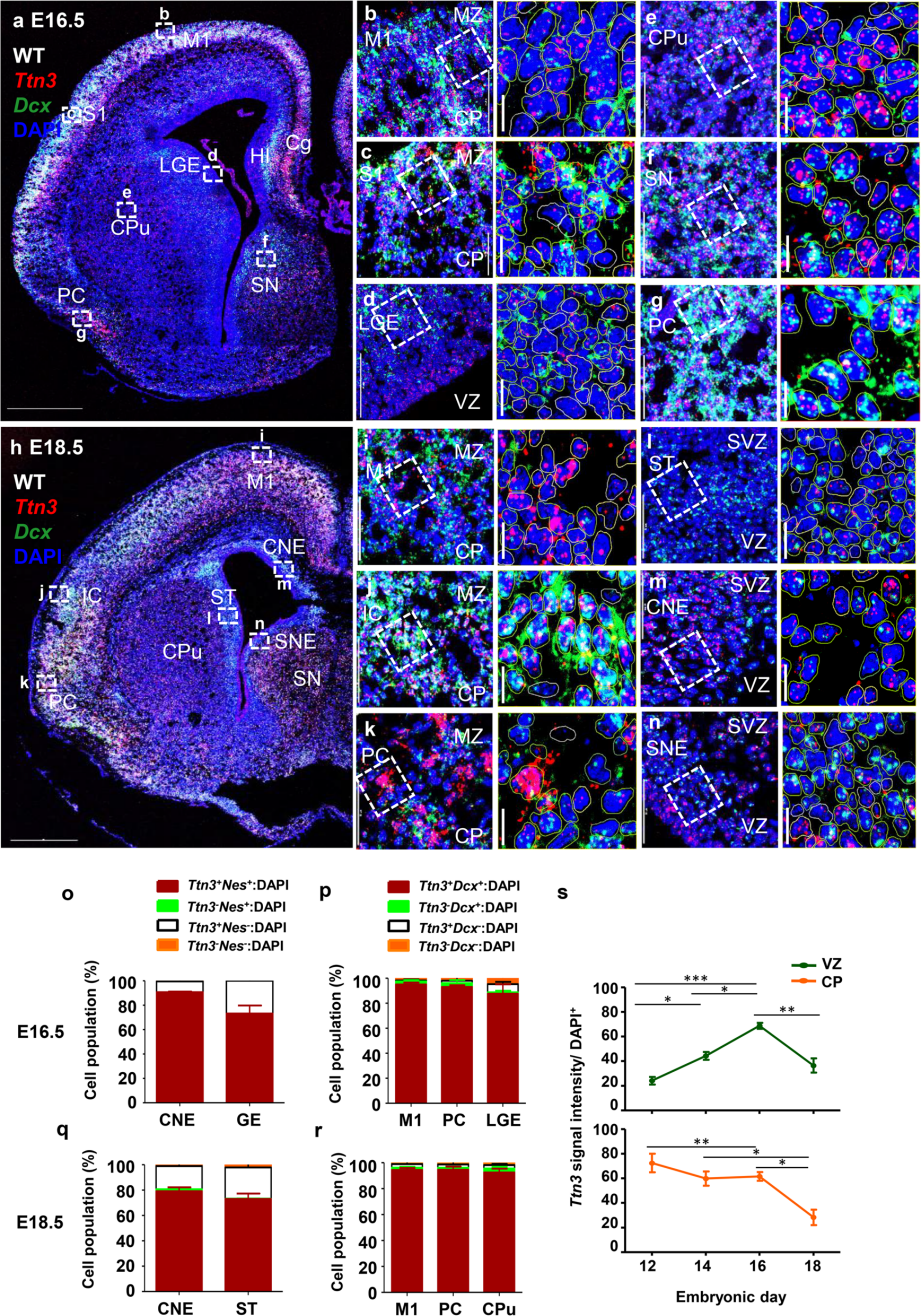
Expression and co-localization of *Ttn3* with *Dcx *in the developing forebrain at E16.5 and E18.5.** a, h** Tile scans of E16.5 (**a**) and E18.5 (**h**) mouse forebrain sections showing *Ttn3* (red) expression, counterstained with DAPI (blue), and co-labeled with *Dcx* (green). Labeled regions include the M1, S1, HI, SN, PC, CPu, Cg, LGE, IC, ST, CNE, and SNE providing an overview of *Ttn3*, and *Dcx* distribution. **b–g, i–n** High-magnification (40 ×) images of specific brain regions. Specific areas imaged include: CP and MZ in M1 (**b**), S1 (**c**), and PC (**g**), VZ in LGE (**d**), IZ in CPu (**e**) and SN (**f**), MZ and CP in M1 (**i**), IC (**j**), and PC (**k**) regions, VZ and SVZ layers in ST (**l**), CNE (**m**), and SNE (**n**) regions. **o–r** Quantitative analysis of cell populations: **o** At E16.5, cells in the VZ of the CNE and GE were classified into four groups: *Nestin⁺: Ttn3*⁺ (red), *Nestin⁺: Ttn3⁻* (green), *Nestin⁻: Ttn3⁺* (white), and *Nestin⁻: Ttn3⁻* (orange). **p** Cells in the CP of the M1, PC, and IZ of LGE were categorized into four groups: *Dcx⁺:Ttn3⁺* (red), *Dcx⁺:Ttn3*⁻ (green), *Dcx⁻:Ttn3⁺* (white), and *Dcx⁻:Ttn3⁻* (orange). **q** At E18.5, cells in the VZ of the CNE and ST were categorized into four groups: *Nestin*⁺: *Ttn3*⁺ (red), *Nestin*⁺: *Ttn3*⁻ (green), *Nestin*⁻: *Ttn3*⁺ (white), and *Nestin*⁻: *Ttn3*⁻ (orange). **r** Cells in the CP of the M1, PC, and IZ of the CPu were classified into four groups: *Dcx*⁺: *Ttn3*⁺ (red), *Dcx*⁺: *Ttn3*⁻ (green), *Dcx*⁻: *Ttn3*⁺ (white), and *Dcx*⁻: *Ttn3*⁻ (orange). **s** Intensity analysis of *Ttn3* expression levels, comparing the VZ and CP across developmental stages in the cortex. (*P < 0.05, **P < 0.01, ***P < 0.001; statistical significance determined by one-way ANOVA followed by Tukey’s post hoc test.). Abbreviation: M1 motor cortex; LGE lateral ganglionic eminence; S1 somatosensory cortex; CPu caudate putamen; SN septal nucleus, PC piriform cortex, ST striatal, CNE cortical neuroepithelium. Scale bars: **a, h** 500 µm; **b–g, i–n** 50 µm; cropped image 10 µm. All quantitative analyses were performed using *n* = 3 mice per group**.** Individual data values are provided in Additional file 4

During the neurogenic period, *Nestin* expression typically decreases as neurons mature, while *Dcx* expression, indicative of neuronal differentiation, becomes more prominent. Previous studies have shown that *Nestin* persists in certain apical RG within the VZ and begins to appear in non-neuronal populations such as oligodendrocyte progenitor cells (OPCs), which migrate from regions like the GE to the cortex [20–22]. In parallel, *Dcx*⁺ immature neurons undergo tangential migration from the subpallium to the pallium, as well as radial migration from the VZ to the CP [23]. These patterns are consistent with our observations of *Dcx* and *Nestin* expression (Fig. 3a–g, Additional file 3: Fig. S3a-f).

Similar to earlier developmental stages, the overall intensity of *Ttn3* was further elevated at E16.5, particularly in the FC, including the PC, somatosensory cortex 1 (S1), and motor cortex 1 (M1, Fig. 3a). Higher-magnification images confirmed *Ttn3* expression in several additional regions, including the LGE, CPu, SN, and hippocampus (HI), with its distribution spanning all brain layers, including the VZ, SVZ, IZ, marginal zone (MZ), and CP (Fig. 3b–g, Additional file 3: Fig. S3b-f). Stronger signals were consistently observed in the IZ compared to the VZ and SVZ, with progressively higher expression in superficial layers such as the CP and MZ (Fig. 3a, Additional file 3: Fig. S3a).

As expected, *Ttn3* was also co-expressed with *Eomes* in the SVZ and other progenitor-rich domains of the cortex and HI (Additional file 1: Fig. S1g-i). Cell population analyses revealed that all *Nestin*⁺ cells in the cortical VZ and GE expressed *Ttn3* (Cortex: 100%, GE: 100%), and *Ttn3* was detected in *Nestin*⁻ cells comprising approximately 8% in the cortex and 21.1% in the GE (Fig. 3o).

In the IZ of CP regions, *Ttn3* was expressed in most of the *Dcx*^+^ cells (M1 95%, PC 93%, LGE 87%), with only a tiny fraction of cells lacking *Ttn3* expression (M1 2.3%, PC 3.1%, LGE 1.1%). In the IZ of LGE, 6.4% of the *Dcx⁻* cells, which constitute 13% of the total cell population, showed *Ttn3* expression (Fig. 3p). These findings imply that *Ttn3* may play comprehensive roles during embryogenesis, pervasively expressed in both progenitors and differentiating neuronal populations at E16.5.

### *Ttn3* expression patterns in E18.5 developing mouse brain

At E18.5, the cortical layers are distinctly organized into the VZ, SVZ, IZ, SP, CP, and MZ. *Ttn3* maintained a similar expression profile as observed at E16.5, exhibiting widespread distribution across various brain regions, including the striatal (ST), caudate putamen (CPu), SN, and cortical areas such as M1, insular cortex (IC), and PC (Fig. 3h). However, with the more refined layer definition at E18.5, *Ttn3* expression appeared more localized, with its highest intensity in the CP (Fig. 3h).

*Ttn3* was detected in both *Nestin*⁺ and *Nestin*⁻ cells across multiple layers, beginning with the VZ and SVZ of the cortical neuroepithelium (CNE, Additional file 3: Fig. S3h), the ST region (Additional file 3: Fig. S3k), and the SNE (Additional file 3: Fig. S3j). Its expression extended through the IZ of the white matter (WM) (Additional file 3: Fig. S3i), CPu (Additional file 3: Fig. S3l), and medial septal nucleus (MSN) (Additional file 3: Fig. S3m). *Ttn3* was also observed in *Dcx*⁺ cells within the CP and MZ of M1, IC, and PC (Fig. 3i–k). Notably, the MSN (Additional file 3: Fig. S3m) and PC (Fig. 3k) exhibited stronger *Ttn3* signals compared to the IZ, CP, and MZ layers in other E18.5 regions, as well as to the same layers at earlier stages. This finding may suggest a differential regulation of *Ttn3*, or it could reflect variations in mechanical properties—such as tissue or cell population stiffness—across different embryonic days and brain regions.

Cell population analysis showed that nearly all *Nestin*⁺ cells expressed *Ttn3*, with approximately 99% in the cortical VZ and 98.2% in the ST neuroepithelium (Fig. 3q). Other cell populations within the VZ also displayed *Ttn3* expression, with about 19.8% of *Nestin*⁻ cells in the cortical VZ and 25% in the ST being *Ttn3*⁺ (Fig. 3q). In the CP region of M1, PC, and the IZ of the CPu, almost all *Dcx*⁺ cells co-expressed *Ttn3* (98% in M1, 97.1% in PC, and 97.1% in the CPu). A small fraction of *Dcx*⁺ cells lacked *Ttn3* (1.7% M1, 1.6% PC, and 3.2% CPu) (Fig. 3r). Overall, these findings suggest the widespread presence of *Ttn3* across diverse cell populations from the VZ to the CP.

Finally, we analyzed cortical intensity to better distinguish *Ttn3* expression differences between the VZ and CP at E12.5, E14.5, E16.5, and E18.5 (Fig. 3s). Overall, the CP consistently exhibited marginally higher *Ttn3* expression than the VZ at each stage, the most significant increase in VZ *Ttn3* intensity was observed from E12.5 to E16.5, correlating with the expanding population of migrating IPs—implying a critical role for *Ttn3* in NSC migration.

### TTN3 deletion and inhibition impair NSC proliferation and migration

**Fig. 4:**
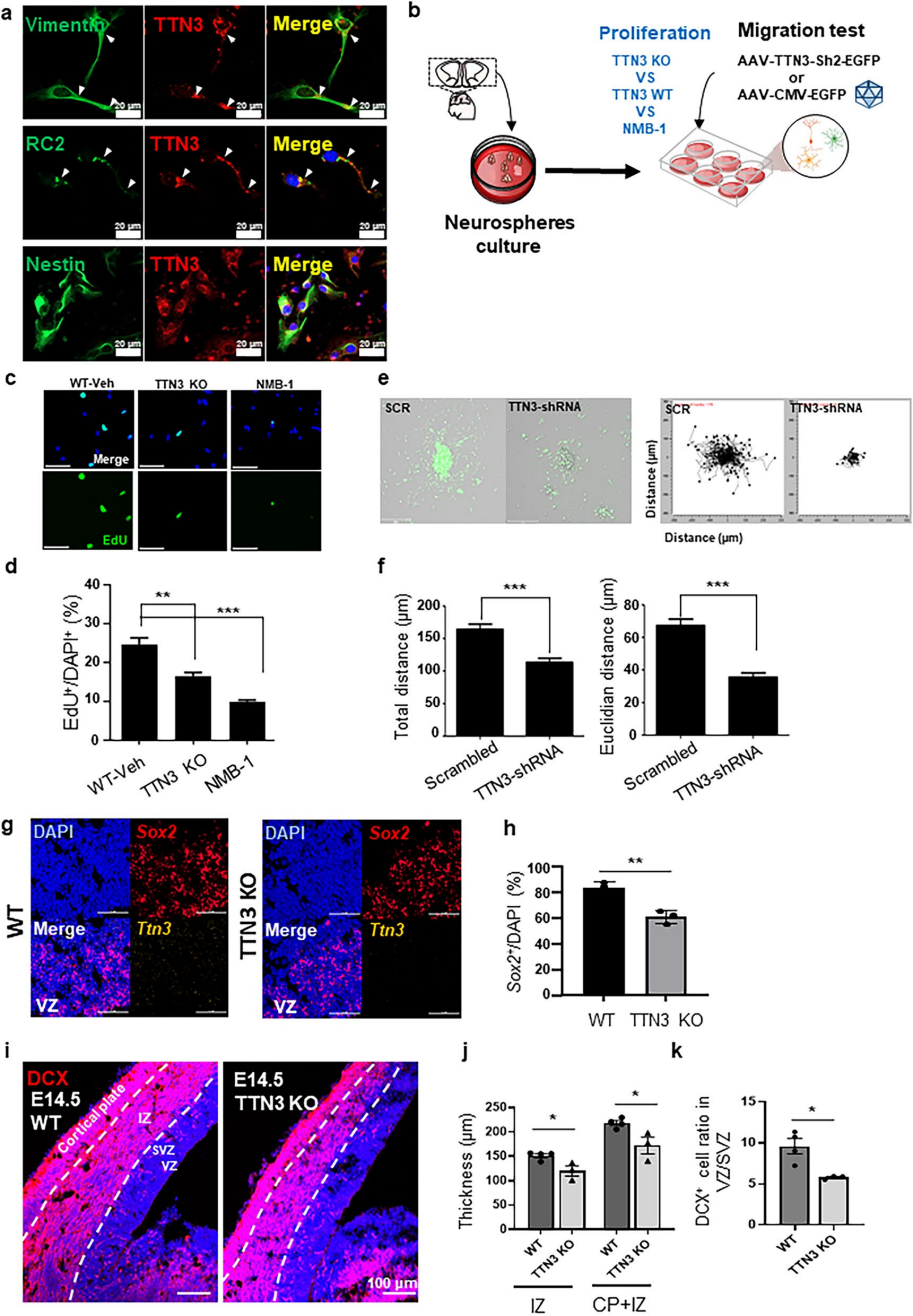
Decrease in proliferation and migration of neural stem and progenitor cells by TTN3 blockage.** a** Representative confocal images of neurosphere-derived cells stained with anti-Vimentin, anti-RC2, or anti-Nestin with TTN3. **b** Schematic overview of the proliferation and migration assay using neurosphere-derived cells. For the proliferation assay, either TTN3 whole KO mouse E14.5 cortex-derived cells or WT mouse cells were used in one group, while in the other group, cells were treated with either NMB-1 or vehicle (DMSO). For the migration assay, mouse E14.5 cortex-derived cells were infected with either scrambled (AAV-U6-SCR-CMV-EGFP) or TTN3-shRNA (AAV-U6-mTTN3-Sh2-CMV-EGFP). **c** Representative images of EdU^+^/DAPI^+^ cells in neurosphere-derived NSCs cultured from WT and TTN3 KO embryos, treated with vehicle (DMSO) or 10µM NMB-1. scale bar 50 µm. **d** Quantification of EdU^+^/DAPI.^+^ cells. (**P < 0.01, ***P < 0.001, student’s t-test) **e** Representative confocal images of neurosphere from the scrambled- and TTN3-shRNA-infected groups for 12 h of the migration assay. **f** Summary of the cell migration. Total migration distance (left) and Euclidean distance (right) were quantified. (*n* = 176 cells from 5 independent cultures for the TTN3-shRNA group and *n* = 88 cells from 3 independent cultures for the scrambled group). **g** Representative mRNA staining images of cortical tissue from WT and TTN3 KO embryos. Scale bar: 5 µm. **h** Quantification of *Sox2⁺*/DAPI (%) in the cortical VZ/SVZ of WT and TTN3 KO embryos. **i** Representative immunohistochemistry (IHC) images of cortical tissue from WT and TTN3 KO embryos. Scale bar: 100 µm. **j** Thickness of the DCX⁺ region in the IZ and CP + IZ of WT and TTN3 KO embryos. **k** Ratio of DCX⁺ cells in the VZ/SVZ of WT and TTN3 KO embryos. Student’s *t*-test, ***P < 0.001. All quantitative analyses were performed using *n* = 3 mice per group**.** Individual data values are provided in Additional file 4

To confirm the presence of TTN3 in NSCs derived from mouse E14.5 cortical tissue, we performed immunostaining using anti-TTN3, anti-vimentin, anti-RC2, and anti-Nestin antibodies. Confocal imaging verified that neurosphere-derived NSCs robustly express TTN3 (Fig. 4a). We next explored the functional role of TTN3 in NSC proliferation and migration by employing both genetic and pharmacological manipulations (Fig. 4b). To assess proliferation, we performed an EdU assay either in the TTN3 whole KO mouse line or in the presence of TTN3 pharmacological inhibition. TTN3 KO led to a pronounced reduction in EdU incorporation compared to WT NSCs, indicating that TTN3 loss significantly impairs proliferative capacity (Fig. 4c, d). To confirm that this effect is linked explicitly to TTN3’s MS channel function, we treated NSCs with NMB-1, a known TTN3 inhibitor [14, 24, 25]. NMB-1 treatment similarly reduced EdU incorporation relative to WT-vehicle controls, paralleling the deficit observed in TTN3 KO NSCs (Fig. 4c, d). Together, these findings demonstrate that TTN3 is positive regulator of NSC proliferation, as both genetic ablation and pharmacological inhibition severely diminish proliferative potential.

Our previous work identified Piezo1 as a stop-signaling mediator that modulates the migration of immature neurons during embryogenesis [8]. Although Piezo1 can also facilitate migration by transducing mechanical forces into electrochemical signals in certain contexts [26], its overall effect in the developing nervous system is to constrain neuronal movement. Based on TTN3’s role as a high-threshold MS channel [14], we hypothesized that it might similarly influence NSC migration.

To test this hypothesis, neurosphere-derived NSCs were plated onto coated dishes, and migration was monitored over 12 h. Fluorescent microscopy revealed a notable difference in migration patterns between TTN3 KD and scramble control groups (Fig. 4e). Control neurospheres exhibited robust outward migration, whereas TTN3 KD neurospheres showed markedly reduced cell dispersal. Quantitative analyses confirmed these observations, demonstrating significantly lower total migration distance and Euclidean distance in TTN3 KD cells than controls (Fig. 4f). These results suggest that TTN3 is required for normal migratory behavior of cells from NSCs and their progeny in vitro.

We next evaluated TTN3’s role in NSC proliferation and migration in vivo using the TTN3 KO mouse line. To assess proliferative capacity, we performed in situ hybridization for *Sox2*, a well-established marker of progenitor cells in the Cortices of VZ/SVZ. *Sox2* expression was robustly expressed in the VZ/SVZ of WT embryos but noticeably reduced in TTN3 KO cortices (Fig. 4g). Quantification confirmed a significant decrease in the number of *Sox2*⁺ cells in TTN3 KO embryos compared with WT littermates, indicating that TTN3 loss attenuates NSC proliferation in vivo, consistent with our EdU assay results (Fig. 4h).

To determine whether TTN3 also regulates neuronal migration during cortical development, we examined DCX expression by immunohistochemistry (IHC) in WT and TTN3 KO embryos. DCX, a canonical marker of migrating immature neurons [27], labels cells as they exit the VZ/SVZ and traverse the IZ toward the CP (Fig. 4i). In TTN3 KO embryos, the thickness of DCX⁺ domains in the IZ and in the CP/IZ region was significantly reduced relative to WT, indicating impaired radial migration (Fig. 4j). In parallel, the proportion of DCX⁺ cells located within the VZ/SVZ was also significantly decreased in TTN3 KO cortices (Fig. 4k), consistent with a reduced production of immature neurons from the progenitor pool and a decreased DCX⁺ cells entering the migratory stream.

Taken together, these in vivo data, combined with our neurosphere assays, demonstrate that TTN3 promotes both NSC proliferation and efficient neuronal migration, supporting its role as an important mechanosensitive regulator of early cortical development.

### Co-expression patterns of *Ttn3* and *Piezo1*

**Fig. 5:**
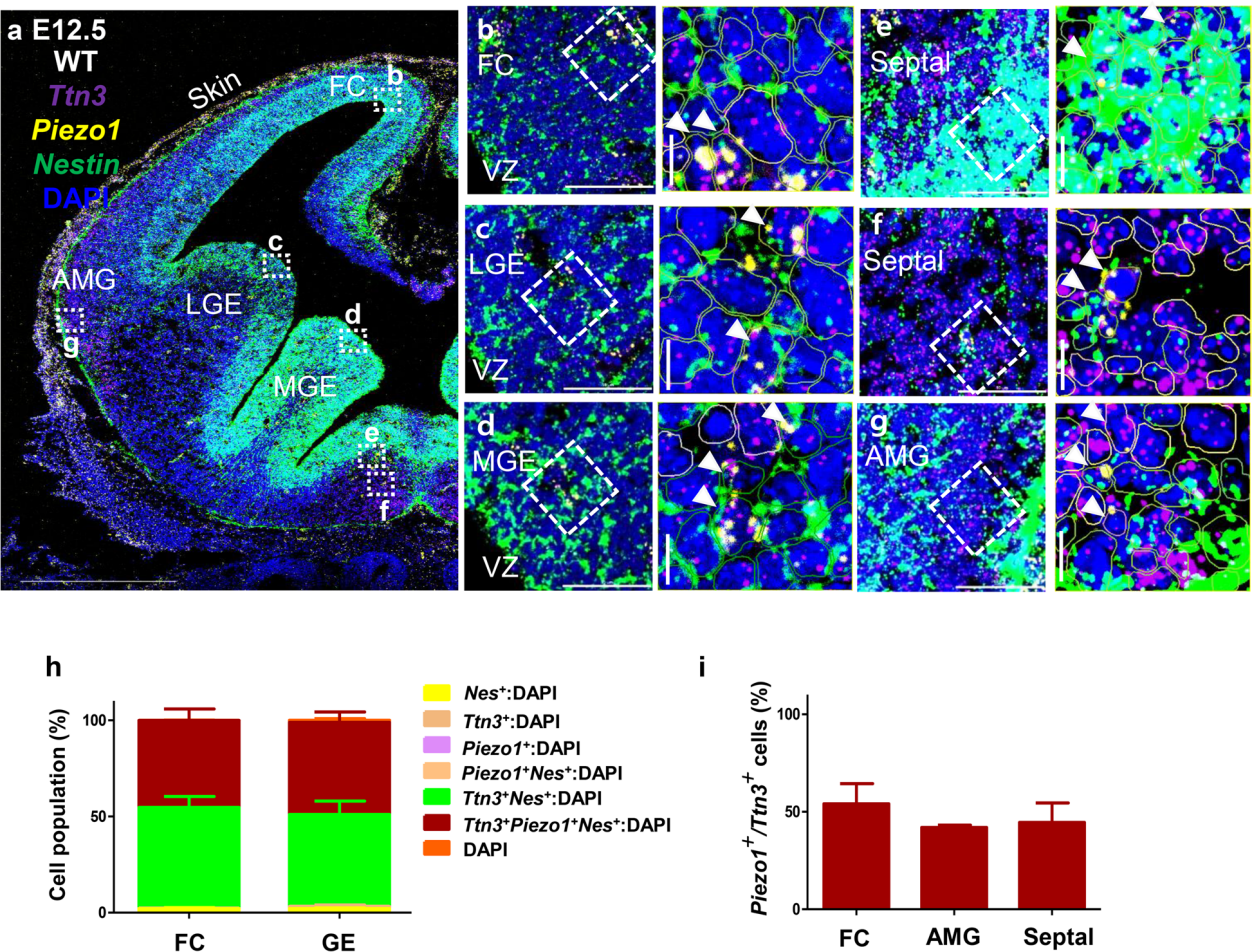
Expression patterns of *Ttn3* with *Nestin* and *Piezo1* during forebrain development at E12.5.** a** Tile scans of an E12.5 mouse forebrain section illustrating *Ttn3* (red) expression with *Nestin* (green) and *Piezo1 (*yellow), co-stained with DAPI (blue) to label nuclei. **b–g** 40 × magnified images of the regions outlined by white boxes in the tile image. The regions include the following: VZ in the FC (**b**), VZ in the LGE (**c**) and MGE (**d**), Septal neuroepithelium region (**e**), Septal IZ layer (**f**), CP in the AMG (**g**). **h, i** Quantitative analysis of cell populations. **h** Summary of cell counts in the VZ of the GE and FC at E12.5. Cell count included: *Nestin*⁺:*Ttn3*⁺:*Piezo1*^+^ (red), *Nestin*^+^:*Ttn3*^+^ (green), *Nestin*⁺:*Piezo1*^+^ (light orange), and singly marked cells, including *Nestin*^+^, *Ttn3*^+^, and *Piezo1*^+^. **i** Summary of *Piezo1*^+^ cells among *Ttn3*^+^ cells in the CP regions of the FC, AMG, and septal at E12.5. Scale bars: **a** 500 µm; **b–g** 50 µm. All quantitative analyses were performed using *n* = 3 mice per group**.** Individual data values are provided in Additional file 4

To explore potential interactions between *Ttn3* and *Piezo1*, we examined their co-expression at E12.5. We conducted co-staining for *Ttn3* (red) and *Piezo1* (yellow), along with *Nestin* (green) (Fig. 5a). *Piezo1* expression was localized to distinct regions, including the VZ, SVZ, and superficial neuronal layers. As confirmed in previous studies, *Piezo1* expression was particularly prominent in the skin and meninges [8], where strong *Nestin* expression was also observed (Fig. 5a). Additionally, longitudinal expression patterns of *Piezo1* were detected across different brain regions, reflecting its involvement in developing vasculature [28]. A detailed imaging revealed co-expression of *Piezo1* and *Ttn3* in multiple regions, including the FC (Fig. 5b), LGE (Fig. 5c), MGE (Fig. 5d), septal region (Fig. 5e, f), and AMG (Fig. 5g). Notably, *Piezo1* levels were higher in the VZ of the septal region than in other areas (Fig. 5e).

Cell population analysis demonstrated that all *Piezo1*^+^/*Nestin*^+^ NSCs in the VZ of the FC and GE were also co-expressed *Ttn3* (cortex: 45.2%, GE: 47.4%; Fig. 5h). In the FC MZ layer, 54% of NSCs were *Piezo1*^+^*Ttn3*^+^, while in the IZ of the AMG and septal region, their proportions were 44.4% and 41.9%, respectively (8 slices, 3 mice; Fig. 5i). These findings indicate that Piezo1 and TTN3 may cooperate in transducing mechanical signals within specific progenitor and neuronal subpopulations during embryogenesis.

## Discussion

NSC proliferation and differentiation are intricately regulated by both chemical and mechanical cues during embryonic brain development [1, 2, 29]. This process demands physiological forces and highlights the potential importance of MS signaling in regulating neural fate decisions [4, 6]. MS channels have emerged as critical mediators of the interplay between the mechanical environment and cell behavior in embryonic brain development. In this study, we investigated the expression patterns of TTN3 and examined its role in NSC proliferation and migration. Our findings indicate that *Ttn3* transcripts are widely distributed across key forebrain regions and developmental time points, with pronounced expression in both *Nestin*⁺ progenitors and *Dcx*⁺ immature neurons. In neurosphere-derived NSCs, TTN3 KD or pharmacological inhibition led to marked reductions in proliferative capacity and migratory distance, suggesting its functional significance.

Mechanotransduction, the process by which cells convert mechanical cues from their niche into biochemical signals, has been shown to regulate diverse aspects of neural development, including cell fate determination, proliferation, and migration [4, 12, 30]. The observed expression of TTN3 in both apical progenitor zones and superficial layers suggests that TTN3 may function throughout multiple stages of embryogenesis. Our intensity analyses revealed that *Ttn3* levels peak around E16.5—when cortical expansion and neurogenic activity are at their height—and then slightly decrease by E18.5. This pattern parallels the dynamic mechanical changes in the developing cortex, such as increased tissue stiffness and evolving ECM composition [4, 30].

Our study provides insight into TTN3’s broad expression in both *Nestin⁺* progenitors from VZ and SVZ. This pervasive distribution suggests a multifaceted role for TTN3 in early corticogenesis, aligning with the notion that mechanotransduction critically governs cell fate and tissue architecture [6, 12]. Our neurosphere experiments demonstrated that TTN3 is critical for NSC proliferation. TTN3 KO or pharmacological inhibition consistently decreased EdU incorporation, indicating that TTN3-mediated mechanotransduction contributes to cell cycle progression. These findings are consistent with prior research showing that mechanical signals can directly influence cell division and progenitor maintenance [12, 30, 31].

Particularly, *Ttn3* expression intensifies in later neurogenic stages, coinciding with heightened neurogenic output and extensive interkinetic nuclear migration (IKNM) in neuroepithelial cells and RGs [2, 3]. Among the diverse mechanical processes in developing brain tissue, IKNM stands out as requiring some of the highest cellular forces, which can be sensed by the high-threshold MS channel, TTN3 [3, 14]. Given the elevated expression of TTN3 in RG and other progenitor cells, it is plausible that TTN3 could sense or modulate these forces, thereby modulating Ca^2^-dependent signaling pathways critical for proper nuclear movement and cell-cycle progression [32]. The slight peak in *Ttn3* at E16.5—when neurogenic output is substantial and nuclear translocation rates are high—further supports the hypothesis that TTN3 may play a role in sustaining these mechanically demanding events.

Our in vitro and in vivo analyses further demonstrate that TTN3 is a positive regulator for efficient NSC migration. This result is particularly compelling since our data demonstrates robust *Ttn3* expression in migrating IPs and immature neurons in regions such as the IZ and CP. Mechanistically, the ability of TTN3 to detect higher-threshold mechanical cues—potentially from ECM stiffness or intracellular forces—may facilitate the directed migration of neuronal precursors as they navigate toward their final laminar positions [14]. Although Piezo1 is another well-characterized MS channel implicated in embryonic brain development, our data and the previous report reveal that Piezo1’s expression is relatively restricted and often associated with distinct neuronal lineages or OPCs [8, 33–35]. Moreover, recent work has suggested that Piezo1 can function as a “stop signal” for migrating neurons, in contrast to TTN3’s apparent role in promoting or sustaining migration [8, 26]. Our co-expression analyses indicate that where Piezo1 and TTN3 overlap, they may act in a complementary fashion, possibly integrating different magnitudes or durations of mechanical forces.

Piezo1 is known to inactivate more rapidly in response to mechanical stimuli, whereas TTN3 exhibits slowly adapting currents [9, 13]. Thus, the two channels may respond to distinct mechanical regimes within the same cellular environment: Piezo1 to acute or transient forces and TTN3 to sustained or higher-threshold stimuli. This dichotomy could help explain the broader and more persistent expression of *Ttn3* we observed across multiple layers and cell types. Future experiments measuring real-time Ca^2^⁺ influx under varied mechanical loads would clarify how TTN3 and Piezo1 integrate mechanical signals during the developing brain.

Several important questions remained for future directions. Firstly, the specific molecular mechanisms by which TTN3 transduces mechanical signals into downstream gene expression or cytoskeletal remodeling warrant detailed investigation. Second, the relative contributions of TTN3 and Piezo1—and potentially other MS channels—to distinct phases of cortical development remain to be fully elucidated. Finally, direct measurements of mechanical forces using atomic force microscopy or other equipment in conjunction with real-time imaging of TTN3 activity would clarify how changing niche stiffness or nuclear migration forces modulate TTN3 function in vivo.

## Conclusions

Our data indicate that TTN3 is a key MS channel in the developing embryonic forebrain, expressed in both progenitors and immature neurons, and crucial for proper NSC proliferation and migration. We propose that TTN3’s slowly adapting properties enable it to respond to sustained or high-threshold forces, such as those encountered during IKNM or within stiffer microenvironments at later embryonic stages. By integrating molecular, cellular, and biophysical approaches, future studies can build on our findings to better understand how MS channels like TTN3 coordinate the complex choreography of neurogenesis, ultimately shaping the architecture and function of the embryonic brain.

## Methods

### Animals and TTN3 KO mice

All animal experiments were conducted in accordance with the guidelines of the Institutional Animal care and Use Committee (IACUC) of the Korea Institute of Science and Technology (KIST) and complied with relevant national regulations for the care and use of laboratory animals. All experimental protocols were approved by the IACUC of KIST (Protocol No. KIST-IACUC-2023–072-2). No anesthesia was used, as all procedures were performed following euthanasia. Pregnant wild-type mice (6–8 months old) were euthanized by gradual-fill carbon dioxide inhalation in a euthanasia chamber. Carbon dioxide was introduced at a flow rate corresponding to 30–50% of the chamber volume per minute, in accordance with the AVMA Guidelines for the Euthanasia of Animals. Death was confirmed by cessation of respiration and heartbeat, after which embryos were immediately collected. Embryos at developmental stages E12.5, E14.5, E16.5, and E18.5 were used in this study.

The *Ttn3* mutant mouse line was generated by the trans-National Institutes of Health KO Mouse Project (KOMP; https://www.komp.org). A LacZ cassette containing a stop codon was inserted between exons 5 and 6 of the *Ttn3* locus, resulting in a truncated *Ttn3*-LacZ fusion product. *Ttn3*^+^/^^^ mice were crossed with wild-type animals to expand the heterozygous colony, and subsequent intercrossing of *Ttn3*^+^/^^^ pairs produced homozygous *Ttn3*^−^/^−^ mice.

### Tissue preparation and fluorescence in situ hybridization

Embryonic brains were collected from euthanized C57BL/6 J pregnant mice at developmental stages E12.5, E14.5, E16.5, and E18.5. The tissues were fixed in 4% paraformaldehyde solution (Biosesang, South Korea) for 24 h, followed by three washes in 1X PBS. Tissue dehydration was then performed sequentially using 10%, 20%, and 30% sucrose solutions in PBS. The tissues were embedded in O.C.T. compound (SAKURA, Japan) and stored overnight at − 80 °C. After embedding, the tissues were sectioned using a cryotome at a thickness of 10–14 µm at − 22 °C and subsequently stored at − 80 °C.

RNAscope was conducted using the RNAScope® Multiplex Fluorescent Detection Reagent Kit v2 (Advanced Cell Diagnostics, ACD, Cat. No. 323100), following the manufacturer’s protocol. Specific RNAscope probes targeting *Ttn3*, *Piezo1*, *Nestin*, and *Dcx* were hybridized for 2 h, followed by fluorescence staining. The tissue sections were then counterstained with DAPI for 5 min. The slides were mounted with antifade reagent (Invitrogen, Cat. No. P36930). Imaging was performed using a confocal microscope (LSM800, Carl Zeiss) with a 20 × objective, capturing z-stack images (5–7 images with 1 µm steps) for whole-brain sections and using a 40 × objective with 1.5 × zoom to capture higher magnification z-stacks (3–4 images with 0.5 µm steps). For cell counting, images were acquired using a 20 × objective with 1.5 × zoom. Cell population counts and intensity analysis were performed using Qupath software. For cell population counts, nuclei were segmented using DAPI in QuPath, and a uniform cytoplasmic expansion (1.5–2 µm) was applied to approximate cell boundaries. RNAscope puncta were detected using QuPath’s subcellular spot detection module with identical thresholds across all images. A cell was classified as probe-positive when it contained one or more discrete puncta, or a puncta cluster larger than 0.4 µm^2^, consistent with ACD’s recommended scoring guidelines. Intensity analysis was performed by consistently selecting comparable regions of interest (ROIs), with signal values normalized to the number of cells within each ROI, or by directly measuring intensity differences between cell types and averaging the values. Both data were further analyzed and represented using GraphPad Prism 5.

### Neurosphere primary stem cell culture

Embryonic brains were collected from euthanized C57BL/6 J pregnant mice at embryonic day 14.5 (E14.5). Following the removal of the skull and meninges, the only the cortex was isolated and dissociated into single-cell suspensions. The cells were then seeded in Ham’s F-12 nutrient mix (Gibco, Cat. No. 11765054), supplemented with B27 (ThermoFisher, Cat. No. 17504044), 1% penicillin/streptomycin (Gibco, Cat. No. 15140122), 20 ng/ml bFGF (Peprotech, Cat. No. 100-18C), and 10 ng/ml EGF (Millipore, Cat. No. 01107). The cultures were incubated in a humidified incubator set at 5% CO_2_, 90% N_2_, and 5% O_2_ for 5–6 days. During dissociation, cells were processed using raw F-12 media.

### Neurosphere migration assay and virus transfection

Neurospheres were gently dissociated to preserve their spherical structure and then seeded onto 100 mm dishes. The spheres were transfected with either an AAV-U6-SCR-CMV-EGFP vector (KIST, #21-A155) as a control or an AAV-U6-mTTN3-Sh2-CMV-EGFP (KIST, #21-A128) vector for *Ttn3* silencing. After 48 h, the neurospheres were dissociated into single cells and seeded into poly-L-lysine-coated 6-well plates. The plates were coated with poly-L-lysine (Sigma, Cat. No. P9155) overnight at 37 °C, washed three times with DPBS, and allowed to dry. Subsequently, the plates were coated with laminin (Gibco, Cat. No. 23017015) for 1 h at 37 °C, washed three times with DPBS, and dried again.

The cells were cultured in F-12 medium supplemented with B27 and 1% penicillin/streptomycin but without bFGF and EGF growth factors to promote differentiation. This differentiation condition was chosen because immature neurons exhibit strong migratory capacity, allowing us to assess migration-driven behaviors more effectively. Imaging of the cells was performed using the EVOS M7000 imaging system (ThermoFisher) with an onstage incubator, capturing one image per hour over a 24-h period. Migration traces were tracked manually using Celleste 6 software.

### HEK293T cell culture and virus transfection

HEK293T (ATCC) were grown in DMEM (Gibco) supplemented with 10% fetal bovin serum (FBS) (Hyclon) and 1% penicillin/streptomycin for two days in a humidified incubator set at 5% CO_2_, 90% N_2_, and 5% O_2_. pIRES2-AcGFP-TTN3 vectors were transfected for 48 h with lipofectamine 2000 (life technologies) or FuGene (Promega).

### EdU proliferation assay

The EdU Proliferation assay was performed using the EdU Proliferation Kit (Abcam, Cat. No. ab219801) according to the manufacturer’s instructions. Briefly, neurospheres were dissociated into single cells through gentle trituration and seeded onto poly-L-lysine and laminin-coated 24-well plates. The cells were cultured in growth factor-depleted F12 media supplemented with B27 and 1% penicillin/streptomycin at a density of 5 × 10^5^ cells/ml on coverslips and incubated for 12 h. For proliferation analysis, cells were treated with 20 µM EdU for 5 h, with the experimental group receiving either 10 µM NMB-1 or vehicle as a control.

Following incubation, the cells were fixed in 1 × formaldehyde, permeabilized with 1 × Triton X-100, and stained according to the manufacturer’s protocol. Nuclei were counterstained with Hoechst 33,342 for 5 min. EdU-positive cells were imaged using the EVOS M7000 imaging system with a 20 × objective. Image analysis was conducted using Celleste 6 software, and the results were visualized using GraphPad Prism 5.

### Immunocytochemistry (ICC)

Neurospheres were dissociated into single cells and cultured on coverslips coated with laminin and poly-L-lysine. The cells were fixed with 4% paraformaldehyde (PFA), permeabilized using 1% Triton X-100 in 0.1% BSA and blocked with 10% serum in PBS. Following these steps, the cells were incubated overnight at 4 °C with primary antibodies against TTN3 (1:500, Millipore), NESTIN (1:1000), VIMENTIN (1:1000, Milipore). The next day, secondary antibodies were applied at 1:800 dilutions and incubated at room temperature for 2 h. Finally, the cells were counterstained with DAPI. Imaging was performed using a confocal microscope (LSM800, Carl Zeiss) with a 63 × objective.

### Tissue preparation and Immunohistochemistry (IHC)

Tissues were collected and fixed in 4% paraformaldehyde (PFA) in PBS at 4 °C for 4 h, followed by cryoprotection in 30% sucrose until fully equilibrated. Samples were embedded in OCT compound and cryosectioned at a thickness of 20 µm using a cryostat. Sections were mounted onto SuperFrost Plus slides and stored at –80 °C until use. Slides were washed in PBS and incubated in blocking solution containing 5% normal goat serum and 0.1% Triton X-100 in PBS for 1 h at RT. Sections were incubated overnight at 4 °C with DCX antibody (1:500, Abcam) diluted in PBS containing 0.1% Triton X-100. After washing with PBS (3 × 5 min), slides were incubated with Alexa 546-conjugated anti-rabbit IgG (1:800, Invitrogen) for 1 h. Sections were counterstained with DAPI (1:500) for 5 min. Images were acquired using a LSM800 confocal microscopy (LSM800, Zeiss) with identical acquisition settings across experimental groups.

### Statistical analysis

All data are presented as the mean ± standard error of the mean (SEM). Statistical analyses were conducted using GraphPad Prism 5. Comparisons between two groups were evaluated using a two tailed Student’s *t*-test. For multiple group comparisons, one-way analysis of variance (ANOVA) followed by Tukey’s post hoc test was employed. A *P*-value less than 0.05 was considered statistically significant (*P* < 0.0001).

## Supplementary Information

Additional file 1: Fig. S1. Expression patterns of *Ttn3* with *Eomes* in E12.5 to E16.5. a, d, g Tile scans of mouse forebrain sections at E12.5 (a), E14.5 (d), and E16.5 (g) showing *Ttn3* (red) expression, counterstained with DAPI (blue) and co-labeled with *Eomes* (green). (b, c, e, f, h, i) Each tile includes higher-magnification insets of the cortex and the medial pallium (MP) region. Scale bars: a,d,g 500 µm; b, c, e, f, h, i 50 µm.

Additional file 2: Fig. S2. Expression patterns of *Ttn3* with *Nestin* in E12.5 TTN3 KO model. a A tile scan revealing *Ttn3* (red) alongside *Nestin* (green) expression patterns in whole TTN3 KO E12.5 developing mouse brain. The dotted boxes are shown in greater details in panel b–g Specific areas imaged include: VZ and CP in the FC (b), VZ in the LGE (c) and MGE (d), Septal region (e), CP in the AMG (f), VZ and CP in the HI (g). Scale bars: a 500 µm; b–g 50 µm.

Additional file 3: Fig. 3. Expression patterns of *Ttn3* with *Nestin* in E16.5 and E18.5. a, g Tile scans of mouse forebrain sections at E16.5 (a), and E18.5 (g) showing *Ttn3* (red) expression, counterstained with DAPI (blue) and co-labeled with *Nestin* (green). b-f, h–m High-magnification (40X) images of specific brain regions, including: VZ in cortex (b), LGE (c), and HI (e), LGE (d), CP in the PC (f), VZ in the CNE (h), WM (i) and SNE (j), IZ in the ST (k), CPu, (l) and MSN (m). CNE: cortical neuroepithelium; LGE: lateral ganglionic eminence; HI: hippocampus; PC: piriform cortex; ST: striatum; WM: white matter; SNE: septal neuroepithelium; MSN: medial septal nucleus; CPu: caudate–putamen. Scale bars: a 500 µm; b–g 50 µm.

Additional file 4. Raw data. Individual data values for all quantitative analyses presented in Figs. 1-5.

## Data Availability

All data generated or analysed during this study are included in this published article and its supplementary information files. The datasets used and analyzed during the current study are available from the corresponding author on reasonable request. The datasets generated during the current study are included in the supplementary information files (Additional file 4).

## Abbreviations

NSCs: Neural stem cells
IPs: Intermediate progenitors
OPCs: Oligodendrocyte progenitor cells
TMEM150C: TTN3: Tentonin 3
MS: Mechanosensitive
ECM: Extracellular matrix
IKNM: Interkinetic nuclear migration
FISH: Fluorescence in situ hybridization
IHC: Immunohistochemistry
Eomes: Eomesodermin
Dcx: Doublecortin
VZ: Ventricular zone
SVZ: Subventricular zone
GE: Ganglionic eminence
LGE: Lateral ganglionic eminence
MGE: Medial ganglionic eminence
CP: Cortical plate
SP: Subplate
IZ: Intermediate zone
MZ: Marginal zone
FC: Frontal cortex
MC: Medial cortex
Cg: Cingulate cortex
PC: Piriform cortex
M1: Motor cortex 1
S1: Somatosensory cortex 1
IC: Insular cortex
CNE: Cortical neuroepithelium
AMG: Amygdala
MP: Medial Pallium
SN: Septal nucleus
SNE: Septal neuroepithelium
LSN: Lateral septal nucleus
MSN: Medial septal nucleus
ST: Striatal
STR: Striatum
CPu: Caudate putamen
HI: Hippocampus
WM: White matter
