# Supplementary figures and images for: Tentonin 3 regulates the proliferation and migration of neural stem cells during embryonic brain development
Source: BMC Biol. 2026 Apr 21;24:133. doi: 10.1186/s12915-026-02604-9 (PMC13231746; doi:10.1186/s12915-026-02604-9)

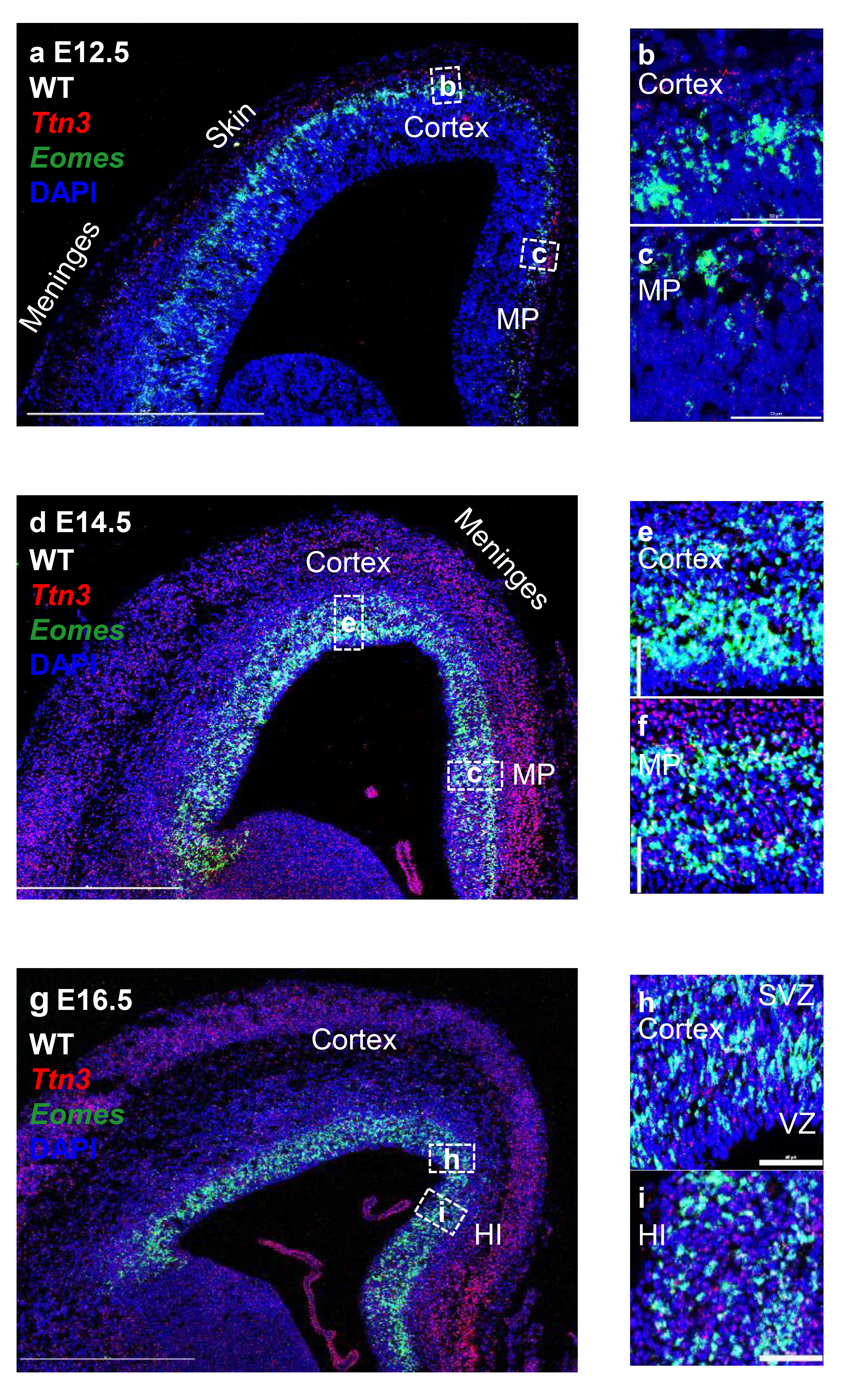

Supplement: Supplementary file 1 — Additional file 1: Fig. S1. Expression patterns of Ttn3 with Eomes in E12.5 to E16.5. a, d, g Tile scans of mouse forebrain sections at E12.5 (a), E14.5 (d), and E16.5 (g) showing Ttn3 (red) expression, counterstained with DAPI (blue) and co-labeled with Eomes (green). (b, c, e, f, h, i) Each tile includes higher-magnification insets of the cortex and the medial pallium (MP) region. Scale bars: a,d,g 500 µm; b, c, e, f, h, i 50 µm. [file 12915_2026_2604_MOESM1_ESM.tif]

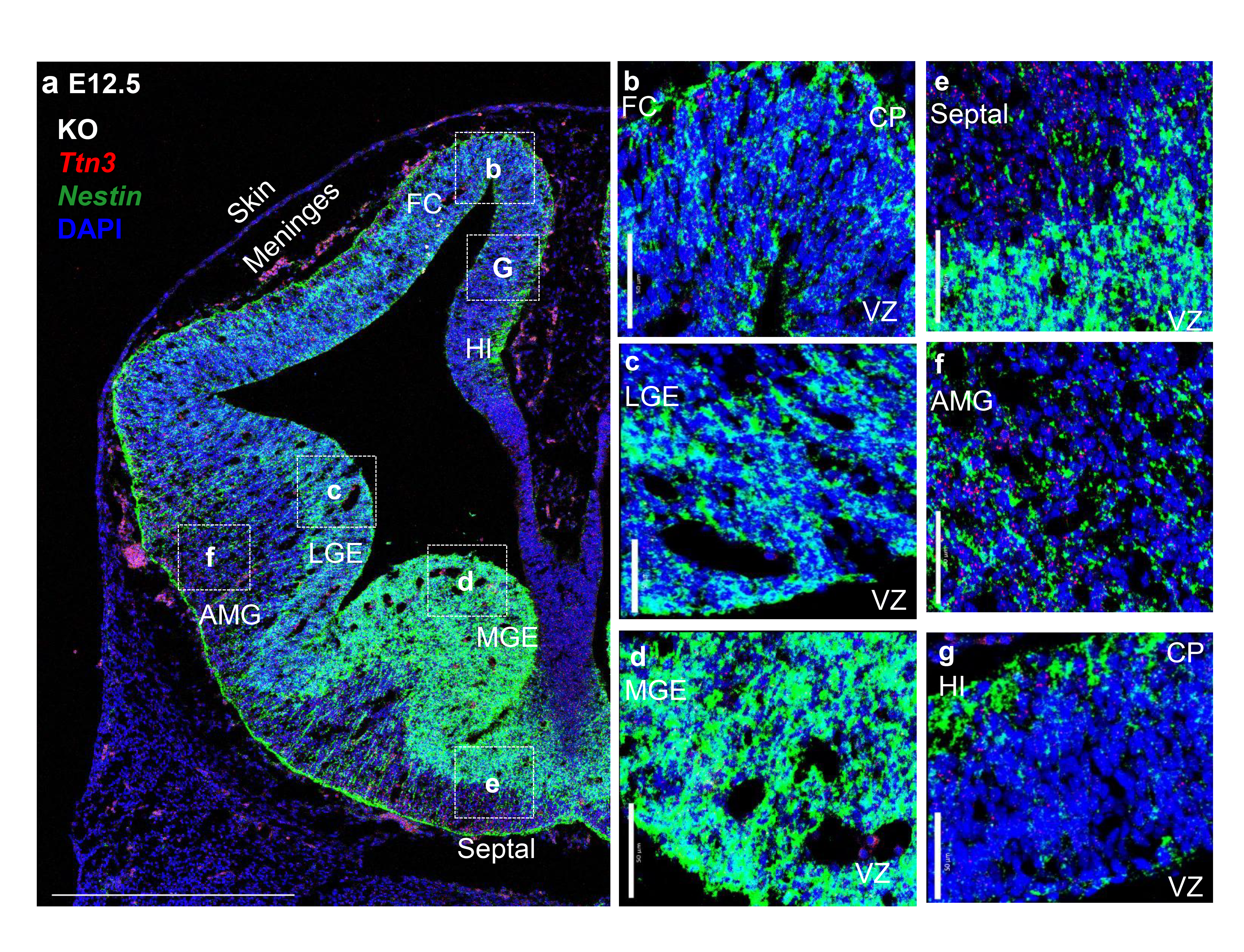

Supplement: Supplementary file 2 — Additional file 2: Fig. S2. Expression patterns of Ttn3 with Nestin in E12.5 TTN3 KO model. a A tile scan revealing Ttn3 (red) alongside Nestin (green) expression patterns in whole TTN3 KO E12.5 developing mouse brain. The dotted boxes are shown in greater details in panel b–g Specific areas imaged include: VZ and CP in the FC (b), VZ in the LGE (c) and MGE (d), Septal region (e), CP in the AMG (f), VZ and CP in the HI (g). Scale bars: a 500 µm; b–g 50 µm. [file 12915_2026_2604_MOESM2_ESM.tif]
